# The Rule of Ultrasonography in the Management of Retained Placenta

**DOI:** 10.7759/cureus.12696

**Published:** 2021-01-14

**Authors:** Rahaf AlMousa, Halah R AlMuhaidib, Sarah M Alanezi, Noura Al Qahtani

**Affiliations:** 1 Obstetrics and Gynecology, Imam Abdulrahman Bin Faisal University, College of Medicine, Dammam, SAU; 2 Emergency Medicine, Imam Abdulrahman Bin Faisal University, College of Medicine, Dammam, SAU; 3 Medicine, Imam Abdulrahman Bin Faisal University, College of Medicine, Dammam, SAU; 4 Obstetrics and Gynaecology, King Fahd Hospital of the University, Khobar, SAU

**Keywords:** retained placenta, postpartum fever, ultrasound, case report

## Abstract

Retained placenta is clinically diagnosed when the placenta has failed to deliver within 18 to 60 minutes after birth. The retained placenta is a risk factor for postpartum fever. In this paper, we report a patient with a delivery complicated by a retained placenta and postpartum fever. This patient, a 34-year-old pregnant female, was admitted at 32 weeks of gestation for a case of preterm labor with preterm premature rupture of membranes and bacterial vaginosis. A 2.5 kg infant was delivered by normal vaginal delivery, which was followed by active management of the third stage of labor. The retained placenta was removed manually under general anesthesia. Two days later, the patient developed a fever and elevated inflammatory markers. Ultrasound-guided evacuation and curettage were done, and two endometrial cavities were noted. Both cavities were evacuated of products of conception. Two days later, the patient started to have spikes of fever. Imaging revealed an intra-cavity soft tissue mass measuring 6.5 cm. Hysteroscopy with dilation and curettage was performed and showed fibrous bands covering a soft mass of products of conception, which was then evacuated.

## Introduction

Retained placenta is a condition that is clinically diagnosed when the placenta has failed to deliver spontaneously within 18 to 60 minutes after birth. In addition, the retained placenta could be considered as a diagnosis if the patient had a hemorrhage preceding the delivery of the placenta. Many risk factors could be attributed to the retained placenta, including but not limited to uterine atony, abnormal adherent placenta such as placenta accreta, history of previous retained placenta, preterm delivery, long use of oxytocin, previous uterine surgeries, and uterine abnormalities. Retained placenta is managed by manual extraction of the placenta, analgesia, and prophylactic antibiotics. If hemorrhaging is present, it should be treated with transfusion and uterine evacuation with suction [1]. It is well known that a retained placenta can result in postpartum fever. In any patient developing a postpartum fever, antibiotic therapy should be initiated. Furthermore, imaging modalities, including ultrasound, must be done for proper evaluation [2]. However, ultrasound is not sensitive and should not be used alone to investigate a case of the retained placenta or postpartum fever [1,2].

In this paper, we report a patient who had a complicated delivery with a retained placenta and postpartum fever.

## Case presentation

A 34-year-old female, gravida 3 para 1+1, was admitted at 32 weeks of gestation to the hospital as a case of preterm labor with preterm premature rupture of membranes and bacterial vaginosis. She had a previous history of preterm labor at 32 weeks, five years prior. In this pregnancy, the patient was on weekly 250 mg progestin injections. She was also admitted five weeks prior with the same presentation, where she received dexamethasone, magnesium sulfate, atosiban, and clindamycin. Two weeks later, the patient was diagnosed with a urinary tract infection and was treated with Augmentin. 

On admission, the patient was vitally stable with progressive cervical dilation. Speculum examination revealed pooling and a positive Amnisure test. Cardiotocography (CTG) revealed a category 1 graph. Ultrasound findings were consistent with gestational age, with an estimated fetal weight of 2 kg. 

The patient was started on azithromycin and ampicillin during the second stage of labor. A 2.5 kg infant was delivered by normal vaginal delivery with an Apgar of nine at one minute and 10 at five minutes. Controlled cord traction was applied with a uterine massage. Ten units of Syntocinon bolus were given, followed by 30 units in 500 mL of normal saline at a rate of 250 mL/hr. 

After 35 minutes, the placenta was not delivered. Upon examination, the placenta was found to be separated, but the cervix was closed. Consent was obtained to take the patient for manual removal of the placenta under general anesthesia. The cervix was held by ovum forceps, and the placenta was evacuated in fragments under ultrasound guidance. A large curette was used on the endometrial cavity. An ultrasound examination revealed a clear endometrial lining. A midline vaginal laceration was repaired and a vaginal back inserted. Two units of PRBCs were transfused, and the patient was started on IV Augmentin and Metronidazole. 

**Figure 1:**
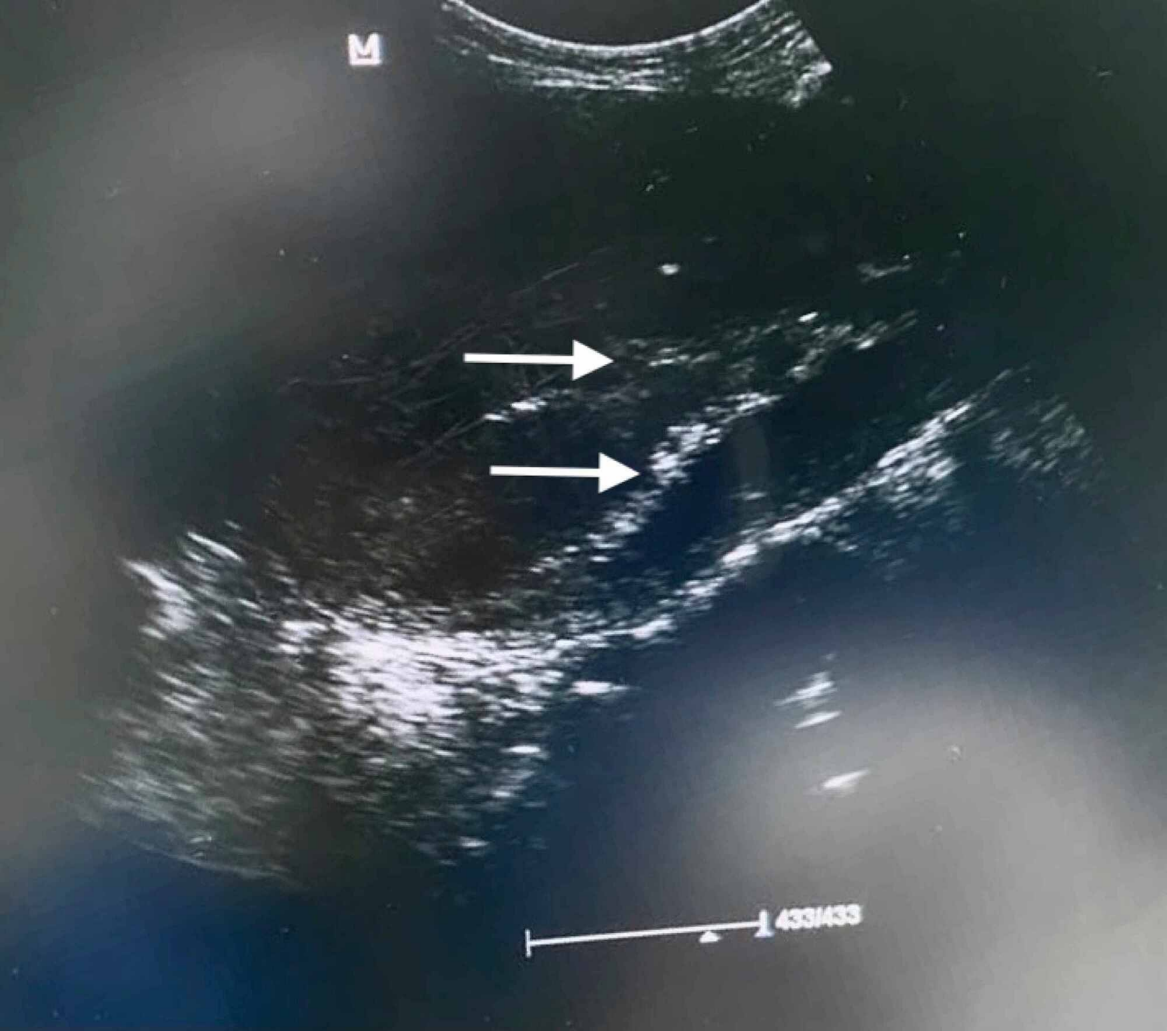
Ultrasound showing two endometrial linings.

Two days later, the patient developed a fever of 38.4°C. Physical examination revealed vaginal bleeding. The vaginal culture was consistent with bacterial vaginosis, along with an elevation of both C-reactive protein (CRP) and erythrocyte sedimentation rate (ESR). Hemoglobin levels continued to drop despite multiple transfusions. However, all other lab tests were unremarkable. The patient was kept on IV augmentin and metronidazole in addition to gentamycin. The patient was shifted to the operation room due to secondary post-partum hemorrhage. Ultrasound-guided dilation and curettage were performed under general anesthesia. Two endometrial linings were noted with what was thought to be a septum (Figure 1). Both cavities were evacuated of the products of conception, and a clear endometrial lining was achieved. 

**Figure 2:**
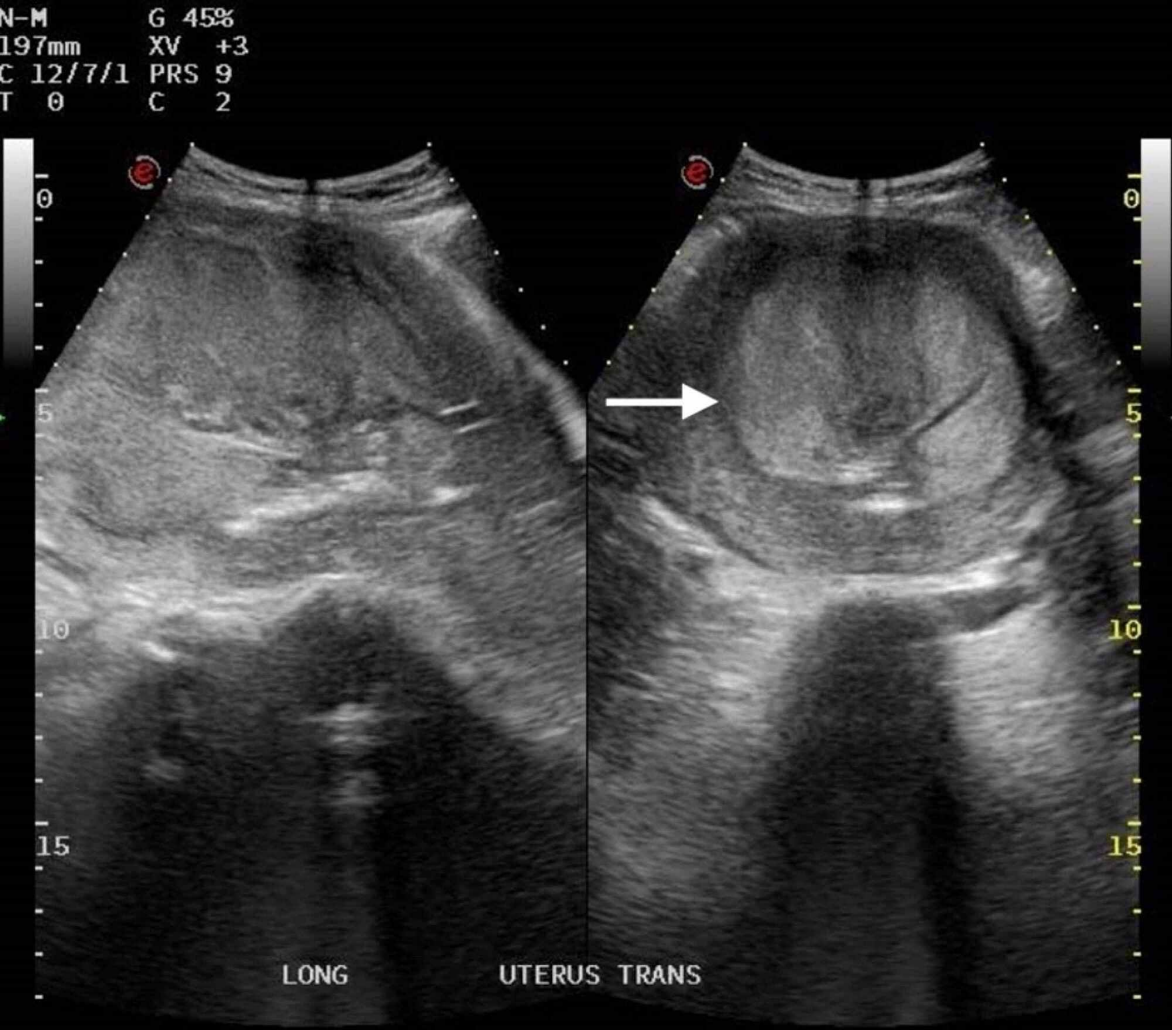
Ultrasound finding of an endometrial mass measuring 10 x 6.6 cm.

Previous antibiotics were discontinued, and the patient was started on tazocin and clindamycin. After two days, the fever kept spiking for 48 hours, the highest measured 38.2°C, alongside an elevation of CRP, ESR, and procalcitonin. Pelvic ultrasound revealed a solid mass of 10 x 6.6 cm with the possibility of an infected product of conception or an infected submucosal/myometrial fibroid with a cervical appearance suggestive of cervicitis (Figure 2).

**Figure 3:**
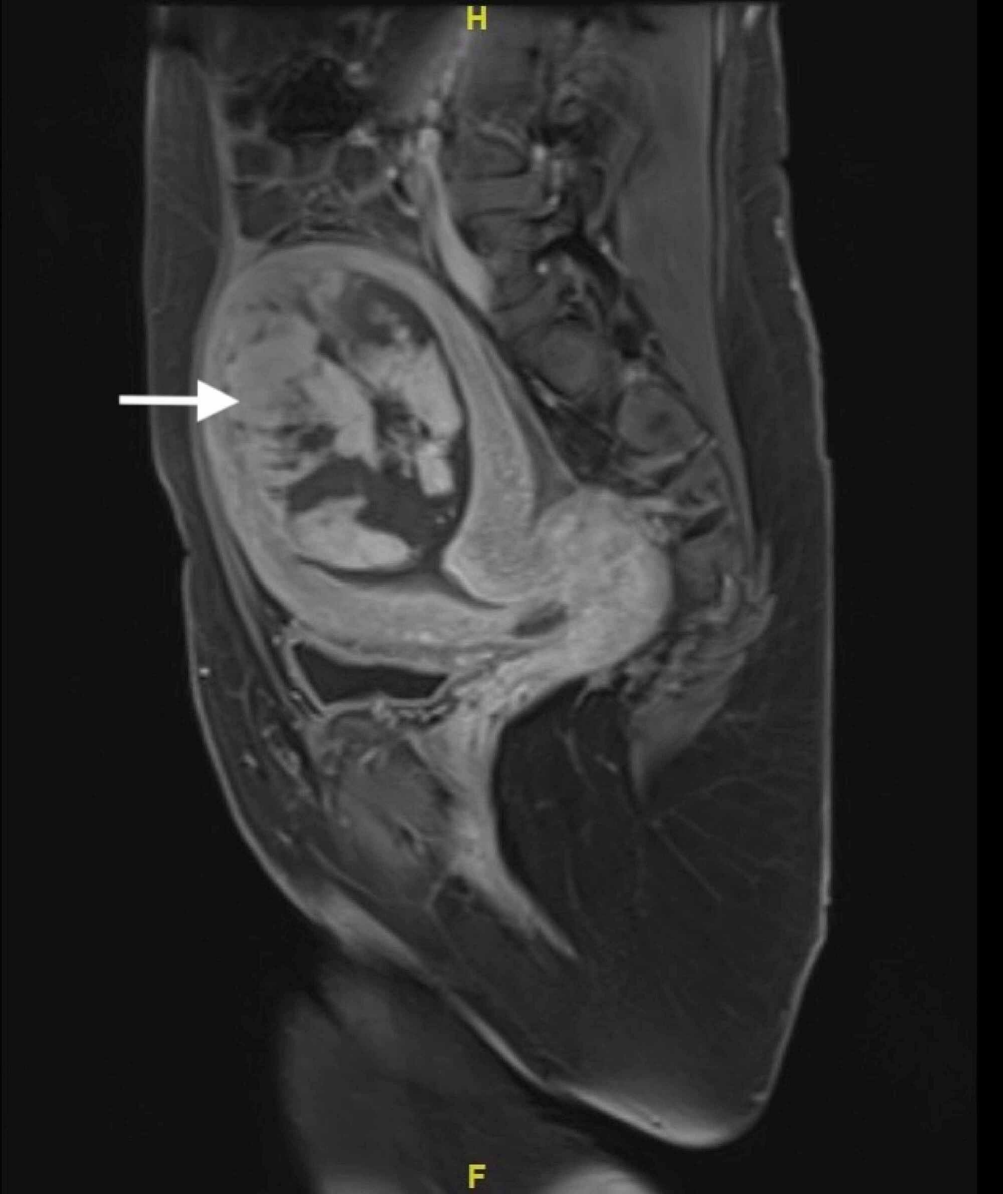
MRI showing an intra-cavity soft tissue mass measuring 6.5 cm.

**Figure 4:**
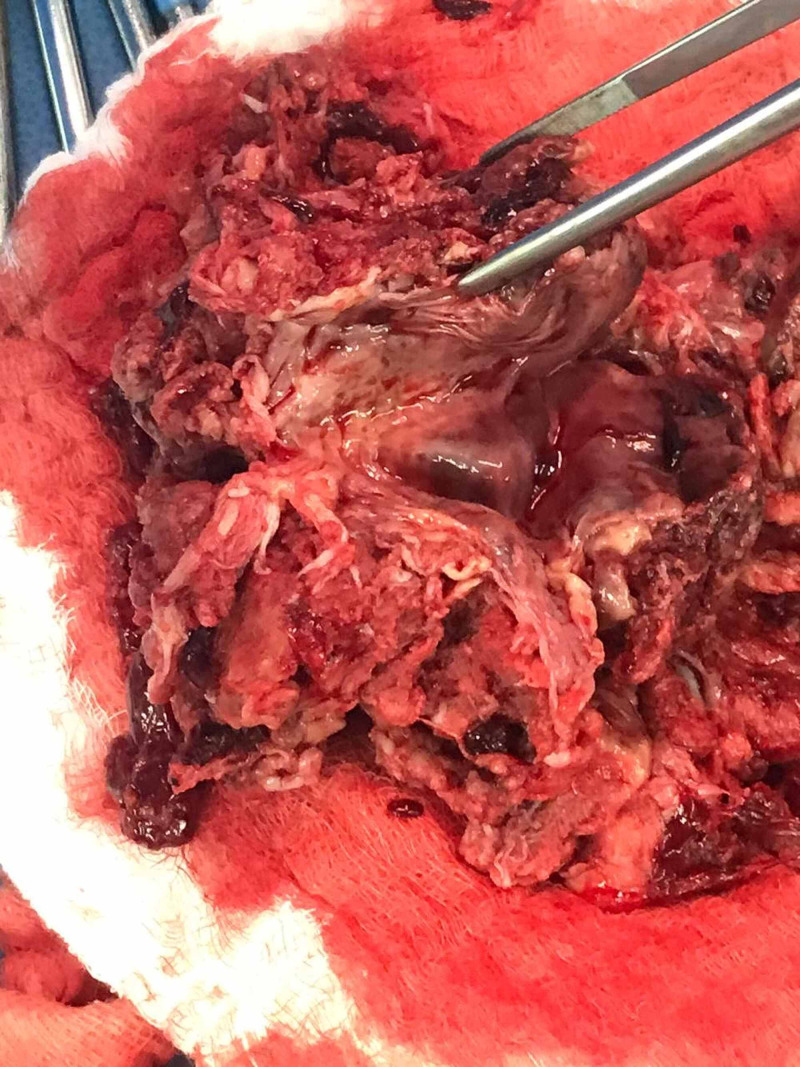
Large products of conceptions, evacuated in segments.

Magnetic resonance imaging showed a single endometrial cavity, which was distended by a soft tissue mass measuring 6.5 cm, with fluids in the pouch of Douglas as well as the vesicovaginal pouch (Figure 3). Consent was obtained to perform a hysteroscopy with dilation and curettage. Upon examination, fibrous bands covering a soft mass were found in the fundus. Once the adhesions were removed, large products of conception were evacuated in segments (Figure 4).

The patient was then started on imipenem post-operatively. The fever subsided, and all the inflammatory markers normalized after evacuation. The patient was discharged after all the symptoms subsided and laboratory parameters normalized. Pathology studies of the uterine contents showed infarcted chorionic villi with massive inflammation and fibro purulent exudate consistent with retained products of conception. Inflamed myometrial tissue was also noted. 

## Discussion

Postpartum fever is a common complication in obstetrics that is often caused by infections introduced during delivery. Postpartum fever can be benign on the first day, especially in primigravida. However, if the fever is equal to or greater than 38°C (100.4°F) and persists beyond one day, management should be initiated [3]. In an observational study done in the United States from 2011 to 2013, using population-based data to explore the most common causes of pregnancy-related mortality in 100,000 births, infections accounted for 12.7%, ranking as the third most encountered cause of mortality. It was followed by hemorrhage at 11.4% [4]. Postpartum pyrexia can also be a nonspecific manifestation of retained products of conception due to infection by cervicovaginal flora; therefore, prompt management should be carried out to eliminate the source of infection and reduce the mortality rate [5]. According to the Royal College of Obstetricians & Gynecologists guidelines, the management of puerperal pyrexia begins with prompt and repeated swabs and blood cultures to identify the microbe and ensure appropriate antibiotic therapy. If sepsis is suspected, the physician should urgently start with parenteral broad-spectrum antibiotics while waiting for the results. Fluid resuscitation and oxygen therapy should be given as they are vital parts of the treatment plan. Serum lactate is an important indicator of tissue perfusion, and a high level >4 mmol/L is indicative of tissue hypoperfusion. Furthermore, in the case of sepsis, serum lactate should be measured every six hours to guide treatment. In addition, imaging modality should be considered to confirm the source of infection [2]. 

Retained placenta is a clinical diagnosis when the placenta does not separate spontaneously at the time of the third stage of labor, or in the scenario of heavy bleeding in the absence of placental delivery. Risk factors include previous retained placenta, previous injury or surgery to the uterus, preterm delivery, induced labor, and multi-parity [1]. Retained placental tissue occurs in 4% of all women [6]. The management of postpartum fever and retained placenta is vital to reduce the mortality of infants and mothers. Focused postpartum ultrasound as a follow-up procedure may not identify every case. In a study involving 172 women, retained placenta cases were more common with vaginal deliveries whose third stage of labor was uncomplicated [7]. Furthermore, the standard management of retained placentas is manual removal under anesthesia, which should be carried out 30-60 minutes postpartum. Other alternatives include medical management of placenta adherents and trapped placentas, such as the use of intra-umbilical oxytocin (30 i.u. in 30 mL saline). As for a trapped placenta, it may respond to glyceryl trinitrate (500 mcg sublingually) or gentle and controlled cord traction [8]. In research done to evaluate sonographic sensitivity for the detection of retained products of conception, the study included 289 patients with clinical suspicion of retained products of conception (RPOC). Sonographic imaging showed 75% sensitivity for the detection of retained placenta, and 25% of these patients underwent unnecessary dilation and curettage (D&C); this is due to the challenging differentiation of retained placenta and other mimics such as blood clots and necrotic material [9].

The patient developed fever two days after delivery in which a full septic workup was taken, including pan cultures, complete blood count (CBC), and inflammatory markers. Despite adequate management with broad-spectrum antibiotics, the fever did not resolve, which led us to further investigate the underlying cause. Pelvic ultrasound (US) and MRI revealed the source of infection. Products of conception were evacuated by hysteroscopy with dilatation and curettage. 

## Conclusions

In conclusion, ultrasonographic-guided D&C is a widely practiced procedure among obstetricians in cases with retained placentas. However, a definitive diagnosis of a clear endometrium should be made with a radiographic modality of higher sensitivity, specificity, and reproducible findings. Close monitoring of the patient’s signs and symptoms, as well as laboratory and radiographic parameters postoperatively, should be considered to aid in the diagnosis and treatment of difficult cases.
